# Distribution and Function of Neuropeptides W/B Signaling System

**DOI:** 10.3389/fphys.2018.00981

**Published:** 2018-07-24

**Authors:** Magdalena Chottova Dvorakova

**Affiliations:** ^1^Biomedical Center, Faculty of Medicine in Pilsen, Charles University, Pilsen, Czechia; ^2^Department of Physiology, Faculty of Medicine in Pilsen, Charles University, Pilsen, Czechia

**Keywords:** NPB, NPW, NPBWR1, NPBWR2, localization, function

## Abstract

Neuropeptide W (NPW) and neuropeptide B (NPB) are two structurally and functionally related regulatory peptides, which are highly expressed in several brain regions and, additionally, in some peripheral tissues. Nevertheless, their distributions in the tissues are not similar. They act on target tissues via two subtypes of G protein-coupled receptors which are designated as NPBWR1 (GPR7) and NPBWR2 (GPR8), respectively, and possess different binding affinities. NPB activates NPBWR1, whereas NPW stimulates both the receptors with similar potency. Both of these peptides takes a part in the central regulation of neuroendocrine axes, feeding behavior, energy homeostasis, cardiovascular functions, circadian rhythm, pain sensation, modulation of inflammatory pain, and emotions. Over the past few years, studies have shown that NPB is also involved in sleep regulation. On the contrary, NPW participates in regulation of vascular myogenic tone, inhibits gastric tension sensitive vagal afferents and insulin secretion. Also, expression of NPW in the stomach is regulated by feeding. Abovementioned findings clearly demonstrate the functional diversity among NPW versus NPB signaling systems. In this review, signal transduction pathways of NPW/NPB are critically evaluated and observed together with mapping of expression of their signaling systems.

## Introduction

Neuropeptide B (NPB) and neuropeptide W (NPW) are peptides with structural and functional similarities. Both are endogenous ligands for G protein-coupled neuropeptide B/W receptors 1 (NPBWR1) and NPBWR2, respectively, and constitute for NPB/NPW signaling systems, which are responsible for regulation of several physiological processes.

Neuropeptide B was independently identified and purified by three different groups from bovine hypothalamus (Fujii et al., 2002; Brezillon et al., 2003; Tanaka et al., 2003). At the same time, Shimomura et al. (2002) had identified NPW from the extract of porcine hypothalamus.

## Structure

**FIGURE 1:**
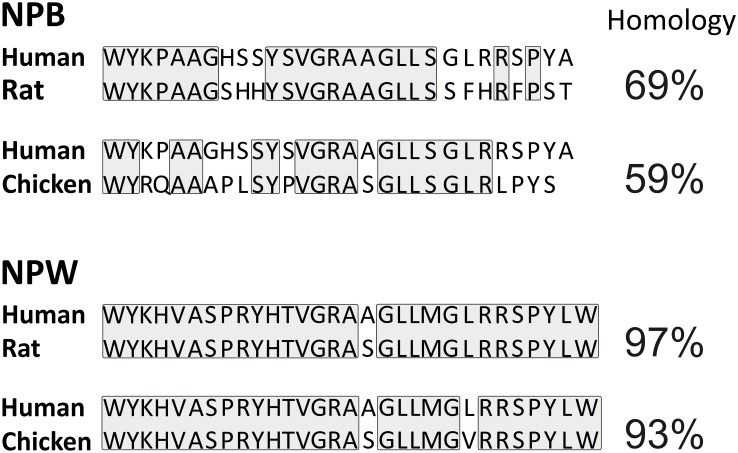
Comparison of amino acid sequence of human, rat, and chicken NPB and NPW peptides. Amino acid residues with identical sequences in compared species are boxed.

Neuropeptide W is present in two isoforms with lengths of 23 (NPW23) and 30 (NPW30) amino acids. Both of them are produced from a common precursor peptide, prepro-NPW by proteolytic cleavage of two pairs of arginine residues at position 24–25 and 31—2 (Takenoya et al., 2010a). The name of NPW is derived from tryptophan residues (single-letter code W) as it appears in both the terminal N- and C- in its two mature forms (Takenoya et al., 2012). Abundance of both these isoforms were demonstrated in several species including human, rat, mouse, pig, and chicken (Brezillon et al., 2003; Tanaka et al., 2003; Kitamura et al., 2006; Fang et al., 2015; Bu et al., 2016). Chicken NPW exerts a noteworthy structural conservation with NPW of mammals (**Figure 1**; Bu et al., 2016).

Neuropeptide B is produced by proteolytic processing from a precursor, prepro-protein called prepro-NPB. In humans, the final peptide sequence of NPB is either shorter (NPB23) or extended (NPB29) which consist of 23 amino acid residues or 29 amino acid residues, respectively (Singh and Davenport, 2006). In non-human species, only NPB29 has been described (Sakurai, 2013). It is unlikely that NPB23 exists in other mammalian species, as the dibasic motif, Arg24–Arg25, used for production of NPB23 is found only in human NPB (Sakurai, 2013). The name for NPB was chosen due to the post-translational bromination at sixth carbon of the indole ring of N-terminal tryptophan (Fujii et al., 2002). Such modification is unique among the mammalian bioactive peptides and its biological significance remains to be clarified, while the brominated and unbrominated forms of NPB exert comparable functional activities (Fujii et al., 2002; Sakurai, 2013). In chicken, NPB precursor produces a mature peptide of 28 amino acids that is different from the 23- or 29 amino acids NPB of mammals (Bu et al., 2016). Additionally, mammalian and avian NPB differ in their N termini where mammals have a “WYK” motif however birds have “WYR” motif. Moreover, chicken NPB shares only 59% amino acid sequence homology with NPB29 of human (**Figure 1**; Bu et al., 2016).

**FIGURE 2:**
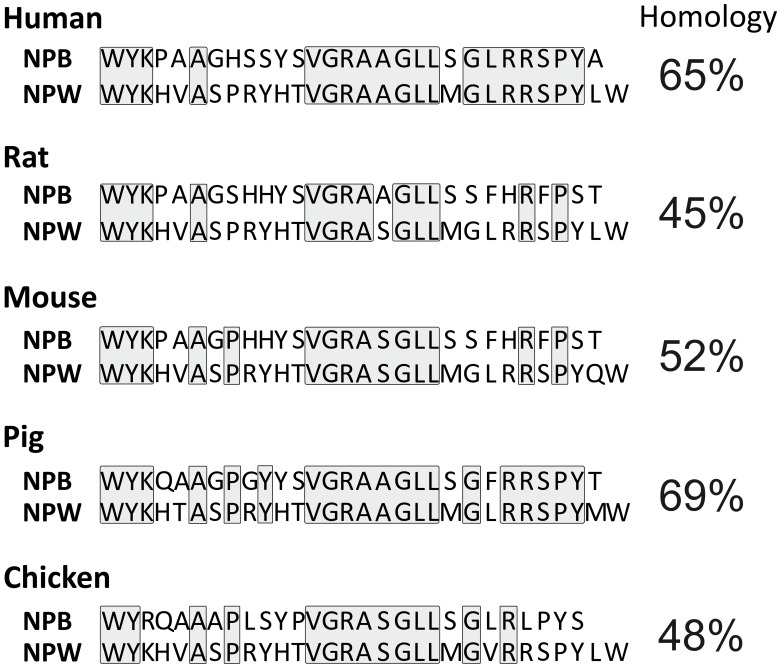
Comparison of NPB and NPW peptides amino acid sequence in human, rat, mouse, pig, and chicken. Identical sequences of amino acid residues are boxed.

Neuropeptide B and NPW do not exhibit any significant sequence analogy to other known peptides but they show a high degree of sequence similarities with each other. NPW23 shows 61% amino acid sequence similarity with NPB23 (Singh and Davenport, 2006), while NPW30 and NPB29 share 65% amino acid sequence similarity in the human. This is analogous to situation in other species as demonstrated in **Figure 2**. Shorter forms of NPW and NPB display similarity at C-terminal secondary structures, while the N-terminal region is more crucial for receptor binding and interaction (Fujii et al., 2002; Tanaka et al., 2003) and differ in their secondary structures (Lucyk et al., 2005). Depletion of Trp1 in either peptide or C-terminally truncated forms of NPB lead to the loss of potency, which suggest that the C- and N-terminus comprise regions of critical amino acids for the binding and efficacy of the peptides to their receptors (Singh and Davenport, 2006).

## Distribution

Several methods were used to determine NPB and NPW mRNA and peptide distribution in different organs and tissues including RT-PCR analysis, *in situ* hybridization, immunohistochemistry, radioimmunoassay, and electron microscopy. Extensive research focused mainly on the expression and distribution of NPB and NPW within the central nervous system (CNS; Fujii et al., 2002; Brezillon et al., 2003; Dun et al., 2003, 2005; Tanaka et al., 2003; Singh et al., 2004; Hochol et al., 2006; Jackson et al., 2006; Kitamura et al., 2006; Schulz et al., 2007; Takenoya et al., 2010b; Fang et al., 2015; Bu et al., 2016), in addition to studies in peripheral tissues (Fujii et al., 2002; Brezillon et al., 2003; Fang et al., 2015; Bu et al., 2016). Both of peptides are often colocalized in different brain structures and the distribution appears not to be species dependent. Differences in findings by immunohistochemistry and *in situ* hybridization could be caused by the very low levels of NPB and NPW mRNAs in some parts of CNS (Takenoya et al., 2010a). There are also some discrepancies between data obtained by immunohistochemistry by different researchers, which are likely due to antibodies from different sources. The CNS distribution of NPB and NPW is summarized in **Tables 1–4**.

**Table 1:** Localization of NPB and NPW in the telencephalon and peripheral nervous system.

	RT-PCR		ISH		IHC		Reference
	NPB	NPW	NPB	NPW	NPB	NPW	
Cerebral hemispheres	C, P	C					Bu et al., 2016; Yang et al., 2018
Hippocampus	Hu, Rt, P	Hu, P	M, Rt	M	Rt	Rt	Fujii et al., 2002; Brezillon et al., 2003; Tanaka et al., 2003; Dun et al., 2005; Kelly et al., 2005; Jackson et al., 2006; Kitamura et al., 2006; Fang et al., 2015; Yang et al., 2018
Amygdala	Hu	Hu	M, Rt	M		Rt, M	Brezillon et al., 2003; Dun et al., 2003; Singh et al., 2004; Kelly et al., 2005; Jackson et al., 2006; Kitamura et al., 2006; Takenoya et al., 2010b; Nagata-Kuroiwa et al., 2011; Motoike et al., 2016
Bed nucleus of the stria terminalis						Rt, M	Kitamura et al., 2006; Takenoya et al., 2010b; Motoike et al., 2016
Septum						Rt	Kitamura et al., 2006
Striatum	Rt					Rt	Fujii et al., 2002
Substantia nigra			Rt				Jackson et al., 2006
Cerebral cortex	Hu, Rt	P, Hu	Rt				Fujii et al., 2002; Brezillon et al., 2003; Jackson et al., 2006; Fang et al., 2015
Corpus callosum	Hu	Hu					Brezillon et al., 2003
Choroid plexus	Hu	Hu					Brezillon et al., 2003
Spinal cord	Hu, C, Rt, P	P, C				Rt	Fujii et al., 2002; Brezillon et al., 2003; Yamamoto et al., 2005; Fang et al., 2015; Bu et al., 2016; Yang et al., 2018
Trigeminal nerve			M				Tanaka et al., 2003
Olfactory bulb	P	P					Fang et al., 2015; Yang et al., 2018
Optic nerve	Hu, Rt						Fujii et al., 2002; Brezillon et al., 2003
DRG	Hu					Rt	Brezillon et al., 2003; Yamamoto et al., 2006

*ISH, in situ hybridization; IHC, immunohistochemistry; Rt, rat; M, mouse; Hu, human; P, pig; C, chicken; DRG, dorsal root ganglia.*

**Table 2:** Localization of NPB and NPW in the forebrain.

	RT-PCR		ISH		IHC		RIA		Reference
	NPB	NPW	NPB	NPW	NPB	NPW	NPB	NPW	
Lateral habenular nucleus			M						Tanaka et al., 2003
Thalamus	Hu, Rt								Fujii et al., 2002; Brezillon et al., 2003
Anteromedial thalamic nucleus						Rt			Kitamura et al., 2006
Dorsal thalamic nucleus			Rt		Rt				Schulz et al., 2007
Hypothalamus	Rt, C, Hu, P	Rt, C, M, P			Rt	Rt		Rt	Fujii et al., 2002; Brezillon et al., 2003; Hochol et al., 2006; Seki et al., 2008; Pate et al., 2013a; Fang et al., 2015; Bu et al., 2016; Wang et al., 2017; Yang et al., 2018
Paraventricular nucleus		Rt	M		Rt	Rt, P			Dun et al., 2003, 2005; Tanaka et al., 2003; Hochol et al., 2006; Takenoya et al., 2008, 2010b; Date et al., 2010; Fang et al., 2015
Supraoptic nucleus					Rt, M	Rt, P			Dun et al., 2003, 2005; Hochol et al., 2006; Schulz et al., 2007; Fang et al., 2015
Periventricular nucleus					Rt				Dun et al., 2005
Suprachiasmatic nucleus					Rt				Dun et al., 2005
Median eminence					Rt	Rt			Dun et al., 2003, 2005; Hochol et al., 2006
Lateral hypothalamic area		Rt	Rt		Rt	Rt, P			Dun et al., 2003, 2005; Jackson et al., 2006; Kitamura et al., 2006; Takenoya et al., 2008, 2010b; Date et al., 2010; Fang et al., 2015
Preoptic area			Rt		Rt				Dun et al., 2005; Jackson et al., 2006
Dorsomedial hypothalamic nucleus			Rt		Rt, M	Rt, P			Dun et al., 2005; Jackson et al., 2006; Kitamura et al., 2006; Schulz et al., 2007; Fang et al., 2015
Ventromedial nucleus		Rt	Rt, M		Rt, M	Rt, P			Kitamura et al., 2006; Schulz et al., 2007; Date et al., 2010; Takenoya et al., 2010b; Fang et al., 2015
Arcuate nucleus		Rt	Rt		Rt, M	Rt, P			Dun et al., 2003, 2005; Jackson et al., 2006; Schulz et al., 2007; Takenoya et al., 2008, 2010b; Date et al., 2010; Fang et al., 2015
Tuberal nucleus			Rt						Jackson et al., 2006
Premammillary nucleus						Rt			Takenoya et al., 2010b
Pituitary gland	Rt, C	Rt, C, P			Rt	Rt			Fujii et al., 2002; Dun et al., 2003, 2005; Hochol et al., 2006; Seki et al., 2008; Fang et al., 2015; Bu et al., 2016

*ISH, in situ hybridization; IHC, immunohistochemistry; RIA, radioimmunoassay; Rt, rat; M, mouse; Hu, human; P, pig; C, chicken.*

**Table 3:** Localization of NPB and NPW in the midbrain.

	RT-PCR		ISH		IHC		Reference
	NPB	NPW	NPB	NPW	NPB	NPW	
Midbrain	C, Rt, P	C, P					Fujii et al., 2002; Fang et al., 2015; Bu et al., 2016; Yang et al., 2018
Colliculi	Hu					Rt	Brezillon et al., 2003; Kitamura et al., 2006
Periaqueductal gray				M, Rt		M, Rt	Tanaka et al., 2003; Singh et al., 2004; Kelly et al., 2005; Kitamura et al., 2006; Takenoya et al., 2010b; Motoike et al., 2016
Dorsal raphe nucleus			Rt	M	Rt	Rt	Tanaka et al., 2003; Singh et al., 2004; Dun et al., 2005; Jackson et al., 2006; Kitamura et al., 2006
Ventral tegmental area				M, Rt	Rt	Rt	Tanaka et al., 2003; Singh et al., 2004; Dun et al., 2005; Kelly et al., 2005; Kitamura et al., 2006; Motoike et al., 2016
Rostral linear nucleus of raphe						Rt	
Interfascicular nucleus					Rt		Dun et al., 2005
Interpeduncular nucleus						Rt	Kitamura et al., 2006
Substantia nigra	Hu	Hu			Rt		Brezillon et al., 2003; Dun et al., 2005
Edinger–Westphal nucleus			M, Rt	M, Rt	Rt	Rt	Tanaka et al., 2003; Dun et al., 2005; Kelly et al., 2005; Jackson et al., 2006; Kitamura et al., 2006
Accessory abducens/facial nucleus						Rt	Kitamura et al., 2006
Hypoglossal nucleus						Rt	Kitamura et al., 2006
Parabrachial nucleus						Rt	Kitamura et al., 2006

*ISH, in situ hybridization; IHC, immunohistochemistry; Rt, rat; M, mouse; Hu, human; P, pig; C, chicken.*

**Table 4:** Localization of NPB and NPW in the hindbrain.

	RT-PCR		ISH		IHC		RIA		Reference
	NPB	NPW	NPB	NPW	NPB	NPW	NPB	NPW	
Hindbrain	C	C							Bu et al., 2016
Medulla oblongata	Rt, P	P						Rt	Fujii et al., 2002; Pate et al., 2013a; Fang et al., 2015; Yang et al., 2018
Inferior olive subnucleus			M						Tanaka et al., 2003
Prepositus nucleus						Rt			Takenoya et al., 2010b
Pons	Hu	P							Brezillon et al., 2003; Fang et al., 2015
Trigeminal nerve nucleus	Hu		M						Brezillon et al., 2003; Tanaka et al., 2003
Parabrachial nucleus			M			Rt			Tanaka et al., 2003; Takenoya et al., 2010b
Locus coeruleus			M, Rt						Tanaka et al., 2003; Jackson et al., 2006
Subcoeruleus nucleus			M						Tanaka et al., 2003
Caudal nucleus	Hu								Brezillon et al., 2003
Cerebellum	Hu, C, Rt, P	Hu, C, P			Rt	Rt			Fujii et al., 2002; Brezillon et al., 2003; Hochol et al., 2006; Fang et al., 2015; Bu et al., 2016; Yang et al., 2018

*ISH, in situ hybridization; IHC, immunohistochemistry; RIA, radioimmunoassay; Rt, rat; M, mouse; Hu, human; P, pig; C, chicken.*

### NPB in the CNS

In the rat CNS, quantitative RT-PCR revealed presence of NPB mRNA in several parts of the brain with highest expression in the hippocampus, hypothalamus, and midbrain (Fujii et al., 2002; Jackson et al., 2006). Evaluations of tissue distribution of the mRNA within the rat brain prove its presence in most areas of the brain. The intensity of staining varies (Fujii et al., 2002; Jackson et al., 2006) suggesting unequal level of transcription of the gene. Immunohistochemical studies detected NPB protein in the nerve cell bodies as well as nerve fibers, although the distribution as well as intensity of labeling differed within the studied parts of the brain. Dun et al. (2005) tested the presence of NPB immunoreactivity (IR) in the hypothalamus, hippocampus, midbrain, and spinal cord, the areas of the CNS with the presence of NPB mRNA. They detect NPR-IR nerve cell bodies in several areas of the hypothalamus and midbrain, but only few of them in the hippocampus, the area of brain with highest NPB mRNA expression. The highest concentration of NPB immunopositive cells was found in the paraventricular and supraoptic nucleus. Strong labeling was detected in the Edinger–Westphal nucleus (EW; Dun et al., 2005). NPB-IR fibers were seen in the medial eminence (Dun et al., 2005; Hochol et al., 2006; Schulz et al., 2007). Both neurons and fibers containing NPB-IR, showed specific IR in the pituitary and cerebellum have been also described (Dun et al., 2005; Hochol et al., 2006). Surprisingly, NPB-IR was not detected in the spinal cord (Dun et al., 2005), whereas presence of NPB mRNA was observed there (Fujii et al., 2002).

Concerning to the human CNS, information about NPB mRNA expression is only available in different parts of the brain. Brezillon et al. (2003) found the greatest NPB mRNA expression in substantia nigra and spinal cord and moderate in hippocampus, amygdala, hypothalamus, corpus callosum, and cerebellum.

In the mouse, study of localization of NPB mRNA shows the strongest expression in the EW, and moderate expression in some nuclei throughout the brain (see **Tables 1–4**) including the hippocampus, hypothalamus, and amygdala (Tanaka et al., 2003; Kelly et al., 2005). Peptide distribution within CNS was studied only in the hypothalamus by means of immunohistochemistry and observed similar staining pattern compared to rat hypothalamus (Schulz et al., 2007).

In the pig, quantitative RT-PCR was used to evaluate NPB mRNA distribution within CNS showing high expression in the cerebellum, moderate expression in the hippocampus, telencephalon, midbrain, and spinal cord, and low expression in the hypothalamus and medulla oblongata (Yang et al., 2018). Additionally, NPB mRNA expression was studied in the chicken tissues including main parts of the CNS by means of quantitative RT-PCR with similar quantities throughout the CNS (Bu et al., 2016).

Studies on mapping expression of NPB in different species show predominant expression of this peptide within the three structures, hypothalamus, hippocampus, and amygdala, suggesting crucial role of the peptide in regulatory processes taking place there.

### NPB in the Peripheral Tissues

In the human, rat, pig and chicken peripheral tissues, NPB mRNA was detected including heart, kidney, urinary bladder, lung, trachea, muscle, ovary, uterus, placenta, testes, prostate, mammal gland, spleen, lymph node, thymus, pancreas, stomach, duodenum, small and large intestine, submandibular gland, liver, white and brown fat, skin, adrenal gland, thyroid gland, pituitary gland, bone marrow, femur, and costal cartilage (Fujii et al., 2002; Brezillon et al., 2003; Bu et al., 2016). Surprisingly, higher levels of NPB mRNA expression compared to CNS were noted in the lymphoid organs of rat and chicken but not in human and pig (Fujii et al., 2002; Brezillon et al., 2003; Bu et al., 2016; Yang et al., 2018). Immunohistochemical experiments demonstrated the presence of the peptide in the anterior and posterior pituitary cells (Dun et al., 2005; Hochol et al., 2006), follicular cells of thyroid gland, all cell types of pancreatic islets, acinar and epithelial cells of the excretory pancreatic ducts, entire adrenal cortex, chromaffin cells of adrenal medulla, ovarial thecal, granulosa and lutein cells and oocytes, Leydig cells of testis, and cells of seminiferous tubules (Hochol et al., 2006, 2007).

As noted above, interspecies differences are more pronounce here compared to nervous tissue. Relatively high expression of NPB mRNA noted in the skeletal muscle in chicken compared to rat, pig, and human suggests some role of this peptide in birds’ skeletal muscle. Nevertheless, this theory remained to be confirmed, while Bu et al. (2016) demonstrated that chicken NPB could activate specific receptor only at high concentrations introducing the question of its functionality in the chicken.

### NPW in the CNS

In the rat brain, RT-PCR and *in situ* hybridization evaluations detected occurrence of NPW mRNA predominantly in different nuclei of the hypothalamus (see **Table 3**) and periaqueductal gray, and in a lesser amount also in the ventral tegmental area, and EW but fail to detect it in the brain cortex (Kitamura et al., 2006; Date et al., 2010; Takenoya et al., 2010b). The NPW protein localization has been minutely studied in the rat brain by several studies showing that NPW-IR distribution within the nervous system is largely similar to that of NPB. NPW-IR neuronal cell bodies were observed in the hypothalamus, pons, ventral tegmental area, periaqueductal gray, EW, and medulla, while NPW-IR fibers were very widely distributed in several regions of the brain including the amygdala, periaqueductal gray, lateral hypothalamus, lateral septal nucleus, and lateral parabrachial nucleus (Kitamura et al., 2006; Date et al., 2010; Takenoya et al., 2010b). Additionally, the presence of NPW peptide in medulla oblongata and hypothalamus was confirmed by radioimmunoassay (Pate et al., 2013a).

In the human CNS, only NPW mRNA but not protein expression was studied yet. The level of expression was highest in the substantia nigra and moderate in the parietal cortex, hippocampus, amygdala, corpus callosum, and cerebellum (Brezillon et al., 2003).

In the mouse brain, *in situ* hybridization method was used to analyze NPW mRNA distribution, which was detected in the hippocampus, periaqueductal gray, ventral tegmental area, EW, and dorsal part of dorsal raphe nucleus (Tanaka et al., 2003; Kelly et al., 2005). Immunohistochemistry revealed presence of NPW peptide in the axon terminals within central amygdala nucleus and bed nucleus of the stria terminalis (Motoike et al., 2016).

In the pig brain, NPW mRNA was detected in several parts of the brain (see **Tables 1–4**) with highest expression in the cerebellum (Fang et al., 2015). Nerve cell bodies as well as nerve fibers containing NPW peptide were present exclusively in the hypothalamus (Fang et al., 2015). In chicken CNS, NPW mRNA was widely expressed (see **Tables 1–4**), while the highest expression was found in the hypothalamus (Bu et al., 2016).

Some studies have focused on characterization of NPB or NPW-IR neurons. NPB-IR neurons in the substantia nigra and ventral tegmental area have been detected to be tyrosine hydroxylase IR (Dun et al., 2005). NPB-IR was also present in a subpopulation of vasopressin-IR cells from the paraventricular, supraoptic, and accessory neurosecretory nuclei but not in the neurons expressing oxytocin (Dun et al., 2005). Motoike et al. (2016) revealed that the majority of NPW neurons in the midbrain are dopaminergic. Additionally, many NPW-IR nerve fibers were in direct contact with melanin-concentrating hormone-containing neurons and orexin-containing neurons in the lateral hypothalamic area (Takenoya et al., 2008). In the dorsal root ganglion, some NPW-IR neurons contained also calcitonin gene-related peptide or isolectin B4 (Yamamoto et al., 2006). In the rat medulla, NPW was co-localized with noradrenaline but not with adrenaline (Seki et al., 2008). Electron microscopic observation revealed that NPW is stored in dense-core vesicles in neuronal axons (Takenoya et al., 2010b).

In the light of abovementioned facts, we can conclude that compared to NPB, NPW is localized in similar parts of the CNS but it is less abundant there. Interspecies differences look to be slight but more comprehensive study is needed to exclude it.

### NPW in the Peripheral Tissues

In the peripheral tissues of pig, NPW mRNA expression has been studied in details and reported to be present in the heart, aorta, esophagus, stomach, small and large intestine, liver, spleen, lymph nodes, thymus, muscle, fat, lung, trachea, kidney, bladder, pancreas, adrenal gland, thyroid gland, submandibular gland, parotid gland, uterus, ovary, testis, epididymis (Fang et al., 2015). Subsequent immunohistochemistry revealed non-neuronal origin of this NPW mRNA, while several cell types exhibit NPW IR including cardiac cells, epithelial cells of gastrointestinal tract, hepatocytes, tubular cells in the renal cortex, oocytes of the primary follicle, follicular epithelial, and parafollicular cells in the thyroid gland and cells in the adrenal gland. Weak NPW-IR was detected in the lymphatic tissue (Fang et al., 2015).

In the rat, NPW mRNA was detected in thyroid and parathyroid glands, pancreatic islets, adrenal gland, testis and ovary, adipocytes, and macrophages (Hochol et al., 2006, 2007; Rucinski et al., 2007; Seki et al., 2008; Skrzypski et al., 2012). Presence of NPW peptide was demonstrated in the follicular and parafollicular cells of thyroid and parathyroid glands, all cell types of pancreatic islets, follicular, acinar, and epithelial cells of the excretory pancreatic ducts, entire adrenal cortex and medulla including small intensive fluorescent extra-ganglionic cells, ovarial thecal, granulosa and lutein cells, and Leydig cells in the testis (Hochol et al., 2006; Rucinski et al., 2007).

In the human, NPW mRNA was demonstrated in the adrenal gland, thyroid gland, pancreas, spleen, lymph node, kidney, prostate, testis, uterus, ovary, placenta, trachea, stomach, rectum, liver, skeletal muscle, and skin (Brezillon et al., 2003), but information of the tissue distribution is still lacking. In the mouse, NPW mRNA expression as well as NPW-IR was detected in the gastric mucosa cells (Li et al., 2013). In the chicken, NPW mRNA was detected in duodenum, pancreas, kidneys, heart, lung, spleen, muscle, ovary, testes, fat, and skin, but not in the liver (Bu et al., 2016).

These results indicate very similar expression of NPW mRNA in peripheral tissues of different species, although the levels of expressions differ between the organs. In the human, the NPW expression is highest in trachea compared to highest expression in pig liver and chicken lung.

## Specific Receptors

Neuropeptide B and NPW act on two specific receptors, NPBWR1 and NPBWR2. Both were identified by cloning of opioid–somatostatin-like receptor genes from human genomic DNA and designated as G protein-coupled receptor 7 (GPR7) and GPR8 (O’Dowd et al., 1995). At that time, their ligands were not identified and they were considered orphan receptors. Later studies discovered that these receptors exert high affinities to peptides NPB and NPW; thus, they had been reclassified by IUPHAR and renamed as NPBWR1 and NPBWR2, respectively (Sakurai, 2013).

The two receptors are functionally coupled to Gi proteins, and their stimulation leads to various intracellular responses in different cell types but also within the cells of the same type. Activation of the receptors may affect the intracellular cAMP level, when the decrease was observed in Chinese hamster ovary cells (Singh and Davenport, 2006), whereas the rise was noted in the human adrenocortical cells (Mazzocchi et al., 2005). Electrophysiology experiments showed different response of hypothalamic neurons to NPW including hyperpolarizing as well as depolarizing effects (Price et al., 2009). In the culture of human adrenal cortex cells, both peptides can activate adenylate cyclase/PKA-dependent cascade but only NPB was able to activate the PLC/PKC-dependent cascade (Mazzocchi et al., 2005). Whereas NPBWR1 is highly conserved in both rodent and human orthologs, NPBWR2 was not found in rodents (O’Dowd et al., 1995; Lee et al., 1999) but has been cloned in several other species, including shrew, rabbit, and lemur. This indicates that there is high conservation of the primary structure, and NPBWR2 gene might have been lost in some mammalian branches (Lee et al., 1999).

In human, NPBWR1 and NPBWR2 share a 70% nucleotide and a 64% amino acid homology with each other. Additionally, they show about 40% amino acid homology with somatostatin and opioid receptors; however, they do not bind somatostatin. NPBWR1 has a low affinity for non-selective opioid ligands (O’Dowd et al., 1995). In mammals, NPBWR1 recognizes both NPB and NPW in nanomolar affinities with moderate preference for NPB, while NPBWR2 is slightly selective for NPW (Tanaka et al., 2003). In birds, NPBWR2 is the only receptor responsible for transmission of information within this signaling system, while NPBWR1 cannot be activated by either of these two peptides in concentrations within physiological range (Bu et al., 2016). This suggests it is lesser or insignificant role in mediating NPB/NPW actions. One possible explanation could be difference in length, while length of NPB is shorter in chicken than in mammals. In rodents, NPBWR2 is lost during speciation (O’Dowd et al., 1995; Lee et al., 1999), so only NPBWR1 is responsible for transmission of NPB/W signals to target tissues.

These findings suggest that mainly NPB signaling system is not well preserved during vertebrate evolution, which may be reflected also in different function of this system in various species. The involvement of various intracellular signaling pathways in response to activation of NPBWR1 or NPBWR2 in different cell types suggests tissue-specific functions of the signaling system.

### NPBWR1 and NPBWR2 in the CNS

In the pig, the expression patterns of NPBWR1 and NPWBR2 are largely similar to each other and also to expression of their ligands (Fang et al., 2015). Lower correlation was found in the human (Brezillon et al., 2003), where NPBWR2 is more widely expressed in the CNS than NPBWR1 but both are highly expressed in hippocampus and amygdala. NPBWR1 is poorly expressed in thalamus, corpus callosum, substantia nigra, and pituitary gland. Immunohistochemical analysis revealed NPBWR1 protein in the myelin-forming Schwann cells in peripheral nerves (Zaratin et al., 2005). Expression of NPBWR2 has been detected in the parietal cortex, caudate nucleus, and cerebellum (Brezillon et al., 2003). In the rat, NPBWR1 mRNA was detected in the taenia tecta, islands of Calleja, olfactory tubercle, primary olfactory cortex, suprachiasmatic nucleus, paraventricular nucleus, supraoptic nucleus, dorsomedial and ventromedial nuclei of the hypothalamus, hippocampus, amygdala, cerebellum, medulla oblongata, and spinal cord (Lee et al., 1999; Fujii et al., 2002). Highest densities of NPW-binding sites were observed in the amygdala, bed nucleus of the stria terminalis, and suprachiasmatic nucleus. Lower levels were detected in the endopiriform nuclei, medial preoptic area, subfornical organ, superior colliculus, periaqueductal gray, and dorsal vagal complex (Singh et al., 2004). The result of detailed study on NPB and NPBWR1 mRNA has partially confirmed the distributions within rat brain and NPB/W-binding sites. The most dense expressions and concentration of binding sites were detected in the amygdala and ventral tuberomamillary nucleus (Jackson et al., 2006). In addition to this, immunohistochemistry has demonstrated the predominant localization of NPBWR1-IR in the neuronal cell bodies and processes of the hypothalamus and neurons of the amygdala. Specific staining was not observed in the cortex or in other cell types, such as glia (Singh et al., 2004) and NPBWR2 does not exist in rodent genome (Sakurai, 2013).

In the mouse nervous system, strong NPBWR1 mRNA expression was found in the amygdala, suprachiasmatic nucleus, paraventricular nucleus, dorsomedial and ventromedial hypothalamic nucleus, periventricular nucleus, insular cortex, zona incerta, anterior part of the dorsal thalamic nucleus, superior colliculus, nucleus of the solitary tract, and dorsal horn of the spinal cord (Schulz et al., 2007; Uchio et al., 2009). NPW-binding sites were present in the amygdala, suprachiasmatic area of the hypothalamus and subfornical organ but not in the cerebellum (Singh et al., 2004). In the pig nervous system, NPBWR1 and NPBWR2 mRNAs were detected in the medulla oblongata, pons, midbrain, hypothalamus, cerebellum, cerebral cortex, hippocampus, hypophysis, olfactory bulb, spinal cord, and nodose ganglia (Fang et al., 2015). In the chicken, NPBWR1 and NPBWR2 mRNAs were detected in the telencephalon, midbrain, hypothalamus, and spinal cord, and NPBWR1 mRNA was also detected in the hindbrain, while NPBWR2 mRNA in the pituitary gland (Bu et al., 2016). The densest expression of NPBWR1 mRNA was detected in the hypothalamus and that of NPBWR2 in the pituitary gland (Bu et al., 2016).

The highest expression of NPB mRNA and the highest amount of NPB-binding sites was found in the amygdala, ventral tuberomamillary nucleus, suprachiasmatic nucleus, and hippocampus, the sites where also dense NPB and/or NPW-IR nerve fibers network is present suggesting functional transmission of NPB/NPW signals to target cells.

### NPBWR1 and NPBWR2 in the Peripheral Tissues

In human peripheral tissues, NPBWR1 and NPBWR2 mRNAs were identified in the testis, lung, large intestine, and skin. NPBWR1 mRNA was additionally detected also in the prostate and trachea, while NPBWR2 mRNA in the adrenal gland, spleen, and lymph node (Brezillon et al., 2003). In the rat, expression of NPBWR1 mRNA was demonstrated in the aorta, mesenteric artery, stomach, large intestine, testis, ovary, uterus, placenta, thyroid gland, adrenal gland, pancreatic islets skin, and adipocytes (Fujii et al., 2002; Hochol et al., 2006; Seki et al., 2008; Skrzypski et al., 2012; Ji et al., 2015). In the pig, both these receptors were detected in several tissues including the heart, abdominal aorta, esophagus, stomach, all parts of the small and large intestines, pancreas, liver, lung, trachea, spleen, jejunal lymph nodes, thymus, muscle, fat, kidney, bladder, submandibular and parotid glands, adrenal gland, thyroid gland, uterus, ovary, testis, and epididymis, although expressions were relatively different in various tissues. Generally, expression of NPBWR1 mRNA in some peripheral tissues was higher in comparison to CNS, such as in the small intestine, pancreas, liver, spleen, muscle, parotid gland, and testis. Similarly, the highest NPBWR2 expression was found in peripheral tissues, such as jejunal lymph node, muscle and fat tissues (Fang et al., 2015). In the chicken, presence of NPBWR1 mRNA was observed in the duodenum and muscle, while NPBWR2 mRNA was detected in the spleen and pancreas (Bu et al., 2016).

In fact, a high number of specific receptors in different peripheral tissues indicate the importance of the NPB/W signaling system in these parts of the body. Especially those with a high degree of expression of these receptors should undergo detailed functional studies targeted for the mapping of their functions here.

## Physiological Effects

### In the Nervous Tissue

A wide distribution of NPB/NPW signaling systems within the CNS suggests several important regulatory functions. Several pharmacological studies indicate their involvement in the regulation of feeding activity, central regulation of neuroendocrine functions, pain sensation, energy homeostasis, autonomic regulation, activation of stress axis, and involvement in the emotions, anxiety, and fear (Sakurai, 2013). It is important to note that some differences in the biological activities between NPB and NPW have been described (Hochol et al., 2004; Mazzocchi et al., 2005; Skrzypski et al., 2012) despite actions on common receptors.

Regulation of feeding and energy metabolism appears to be primary function of the NPB/NPW signaling system, which is complex and include regulation of energy intake as well as endocrine functions involved in metabolism. NPB/NPW involvement occurs at both central and peripheral levels. Within the CNS, NPB- and NPW-containing neurons were observed in hypothalamic areas essential for feeding behavior and energy metabolism. Chronic intracerebroventricular application of NPW led to reduced food intake and weight loss in rats (Mondal et al., 2003). Several functional studies suggest contribution of NPW in energy metabolism, where it probably plays a compensatory role under some circumstances (Date et al., 2010). NPW regulates feeding in complex way, while it acts also in the periphery, where it reduces the sensitivity of tension sensitive gastric vagal afferents (Li et al., 2013). Effects of NPB administration on feeding appears to be doses dependent, where low doses caused mild orexigenic action followed by anorexia, and higher doses induced anorexia at all time points (Ishii et al., 2003; Tanaka et al., 2003). NPB involvement in the maintenance of body weight was demonstrated on NPB gene knockout mice with late onset of obesity (Ishii et al., 2003). While these mice did not significantly differ compared to their littermate controls in either food consumption or activity levels, the authors have suggested that change in metabolic rate is one of the potential explanation of weight differences in mice (Kelly et al., 2005). Involvement of NPBWR1 in this pathway was demonstrated by deletion of the receptor with consequent development of hyperphagia and obesity (Ishii et al., 2003). NPB and NPW also affect production of hormones involved in energy homeostasis. In the pancreatic islet cells, NPW but not NPB perform a potent suppressive effect on blood insulin and leptin concentrations (Rucinski et al., 2007). Both peptides increased cortisol secretion in cultured human adrenocortical cells, although only NPW exerted similar activity on the rat adrenocortical cells (Hochol et al., 2004; Mazzocchi et al., 2005). Interestingly, NPB and NPW directly influence function of adipocytes, as these cells express NPBWR1 (Skrzypski et al., 2012). Long term exposure of adipocytes to NPB and NPW decreased expression of mRNA for leptin. While the two peptides could inhibit food intake as well as leptin secretion, they may alleviate leptin resistance, which is commonly associated with obesity (Enriori et al., 2006; Skrzypski et al., 2012). Data from experiment with leptin deficient and leptin receptor deficient mice suggest an important role of NPW in the modulation of energy metabolism via leptin (Date et al., 2010). Additionally, NPB and NPW have been shown to stimulate lipolysis in isolated adipocytes. NPB, but not NPW, enhances resistin expression and secretion (Skrzypski et al., 2012). Resistin plays important regulatory roles in several biological processes including insulin resistance, diabetes, non-alcoholic fatty liver disease, atherosclerosis and cardiovascular disease, autoimmune disease, asthma, malignancy, inflammatory bowel disease, and chronic kidney disease (Jamaluddin et al., 2012). Involvement of NPB in resistin regulation suggests the potential roles of this peptide in various pathophysiological processes mentioned above.

By contrast, Beck et al. (2010) have demonstrated that NPW does not play primary role in the short-term regulation of feeding behavior, but it could be involved in the long-term interactions between feeding and stress. Involvement of NPB/NPW signaling in the regulation of neuroendocrine system was observed by several investigators on both, central and peripheral levels. Central administration of NPW led to elevation of plasma concentrations of prolactin and corticosterone, and lowering of growth hormone release, the three components of the stress response in rats (Baker et al., 2003). Additionally, the behavioral responses observed mirror those reported in behavioral models of stress (Baker et al., 2003). The localization of members of NPB/NPW signaling system in the key brain nuclei related to stress response also supports the role of this system in stress (Jackson et al., 2006). NPW has been reported to activate the hypothalamic–pituitary–adrenal (HPA) axis via action on the parvocellular paraventricular nucleus of the hypothalamus (Taylor et al., 2005). As mentioned above, both peptides can also regulate activity of peripheral parts of HPA stress axis and adrenal gland (Hochol et al., 2004; Mazzocchi et al., 2005).

Hypothalamic paraventricular nucleus neurons are known to project directly to the sympathetic preganglionic neurons in the spinal cord and control sympathetic outflow (Sawchenko and Swanson, 1983). Intracerebroventricular, but not intravascular, administration of NPW increased mean arterial pressure (MAP) and heart rate (HR), which indicates that NPW produces tachycardic and pressor responses on the central level (Yu et al., 2007). Pate et al. (2013b) demonstrated that such increase in MAP and HR is associated with increased motoric activity of rats after the NPW application and suggests secondary origin of cardiovascular parameter change, while such change was absent in anesthetized animals. Therefore, changes in cardiovascular parameters are probably not caused by direct effects of NPW on hypothalamic paraventricular nucleus neurons and/or sympathetic preganglionic neurons, but more likely mediated by an impact of NPW on orexin neurons in the lateral hypothalamic area, where anatomical evidence for a possible interaction of the two peptides was reported (Takenoya et al., 2008; Pate et al., 2013b). NPBWR1 gene knockout mice keep HR in a basal state and normal blood pressure (Sakurai, 2013). Yu et al. (2007) speculated that NPW could play a role in obesity-related hypertension.

Neuropeptide W expressing neurons localized in the midbrain send projections to the central nucleus of amygdala and bed nucleus of the stria terminalis, which are important for emotive reactions, and express NPBWR1 mRNA (Kitamura et al., 2006). This suggests possible regulatory roles of NPW signaling system in signal transmission between amygdala and other parts of the CNS (Sakurai, 2013), such as the brainstem and bed nucleus of the stria terminalis, which are important for emotion-related autonomic and neuroendocrine responses. NPB/W signaling system could modulate amygdala function in multiple pathways throughout some of its GABAergic interneurons, which also express NPBWR1. Activation of the NPB/W system could cause inhibition of some of the projection neurons in the amygdala, while other projection neurons might be disinhibited through inhibition of GABAergic interneurons (Nagata-Kuroiwa et al., 2011). Dysfunction of the signaling system may result in exaggerated autonomic/neuroendocrine responses along with impaired behavioral response, while NPW gene knockout mice behaved abnormally during and after exposure to novel environmental stimuli (Nagata-Kuroiwa et al., 2011). Motoike et al. (2016) detected inhibition of neuronal activity in the amygdala and bed nucleus of the stria terminalis normally connected with stressful situation in wild type mice was absent in NPW gene knockout mice. They concluded that appropriate NPW signaling is essential for the adequate expression of normal species specific behaviors in response to stressful stimuli. Watanabe et al. (2012) demonstrated in human that genetic differences in NPBWR1 affect emotional responses to facial expressions. Differences in valence evaluation and in dominance rating in seeing angry faces was also described (Watanabe et al., 2012), which suggest involvement of NPBWR1 in social interaction.

The NPB/NPW signaling system is also present in several brain regions linked to pain processing, which is suggestive of a role in modulation of pain transmission. NPB gene knockout mice respond differently to specific types of pain, where they exhibit hyperalgesia in response to acute inflammatory pain, but not thermal or chemical pain (Kelly et al., 2005). Intrathecal administration of either NPB or NPW23 diminished mechanical allodynia via activation of NPBWR1 receptors; nevertheless, the level of thermal hyperalgesia remains stable. These effects were not inhibited by the naloxone, an opioid receptor antagonist, which indicate involvement of an independent analgesic pathway compared to the opioid peptides (Yamamoto et al., 2005, 2006) in which myelin-forming Schwann cells could be involved, while they express low level of NPBWR1 under physiological conditions and in much higher amount in patients with inflammatory neuropathies (Zaratin et al., 2005). Thus, NPB/W signaling can play a role in modulation of nociceptive transmission in peripheral nerves.

Involvement of NPB/NPW signaling system in the regulation of circadian rhythm is indicated by presence of NPW-binding sites in suprachiasmatic nucleus (Singh et al., 2004) and NPBWR1 mRNA expression, which undergoes rhythmical changes in a circadian fashion with a peak in subjective night (Uchio et al., 2009). Surprisingly, both NPB and NPW were not found in the suprachiasmatic nucleus and nearby nuclei (Tanaka et al., 2003; Jackson et al., 2006). Additionally, NPBWR1 gene knockout mice did not exert any abnormality in their basal pattern of sleep/wakefulness (Uchio et al., 2009; Hirashima et al., 2011). Hirashima et al. (2011) showed that administration of NPB in the dark period induced slow wave sleep and concluded that NPB with NPBWR1 may modulate the occurrence of sleep–wakefulness cycle.

### In the Peripheral Tissues

It is difficult to review the central and peripheral effects of these peptides, as their peripheral actions often involved activity in the CNS. In the peripheral tissues, NPW/NPB signaling system regulates vascular smooth muscle tone, endocrine glands activity, metabolism of adipocytes, and stomach activity (Hochol et al., 2004; Mazzocchi et al., 2005; Yamamoto et al., 2006; Rucinski et al., 2007; Skrzypski et al., 2012; Li et al., 2013; Ji et al., 2015).

An important source of plasma NPW is likely from gastric G cells, which produce and release the peptide according to the level of satiety. Lower production occurs during fasting in rats, which is increased after refeeding suggesting important role of NPW in feeding regulation in peripheral level in addition to central regulation (Mondal et al., 2006; Caminos et al., 2008). Li et al. (2014) suggested local role of nutrients to NPW production in the stomach. They show that different nutrients have differential effects on NPW production via various pathways (Li et al., 2014). Additionally, expression of NPW is negatively regulated by thyroid hormones and glucocorticoids in the stomach mucosa (Caminos et al., 2008).

As it has been mentioned above, the NPB/NPW signaling system is expressed in the hypothalamus and several endocrine glands, including pancreatic islets, thyroid and parathyroid gland, adrenal gland, ovary, and testis (Hochol et al., 2006). This suggests possible roles of the signaling system as endocrine system regulators. Until now, there is fragmented information on the roles of this system in endocrine glands. Current data on effects of NPB and NPW on the endocrine glands are summarized in **Table 5**.

**Table 5:** Role of NPB and NPW in regulation of endocrine functions.

	NPB	NPW	Reference
Receptors affinity	NPBWR1 > NPBWR2	NPBWR2 > NPBWR1	Tanaka et al., 2003
Endocrine function			
Basal corticosterone secretion “*in vitro*”	- (r)	↑ (r)	Hochol et al., 2004
Basal cortisol secretion “*in vitro*”	↑ (h)	↑ (h)	Mazzocchi et al., 2005
Basal aldosterone secretion “*in vitro*”	- (r, h)	↑ (r); - (h)	Hochol et al., 2004; Mazzocchi et al., 2005
ACTH stimulated aldosterone secretion “*in vitro*”	↑ (r)	↑ (r)	Hochol et al., 2004
Plasma concentration of corticosterone “*in vivo*”	↑ (r)	↑ (r)	Baker et al., 2003^∗^; Hochol et al., 2006^∗^
Plasma concentration of insulin	– (r)	↓ (r)	Hochol et al., 2006^∗^; Rucinski et al., 2007^∗^
Plasma concentration of thyroxin	↑ (r)	– (r)	Hochol et al., 2006^∗^
Plasma concentration of parathyroid hormone	↑ (r)	↑ (r)	Hochol et al., 2006^∗^
Plasma concentration of testosterone	↑ (r)	↑ (r)	Hochol et al., 2006^∗^
Plasma concentration of estradiol	– (r)	↑ (r)	Hochol et al., 2006^∗^
Leptin secretion from adipocytes “*in vitro*”	↓ (r)	↓ (r)	Skrzypski et al., 2012
Plasma concentration of leptin	– (r)	↓ (r)	Rucinski et al., 2007^∗^
Plasma concentration of ACTH	– (r)	↑ (r)	Hochol et al., 2006^∗^
Plasma concentration of prolactin	n.d.	↑ (r)	Baker et al., 2003^∗^
Plasma concentration of growth hormone	n.d.	↓ (r)	Baker et al., 2003^∗^

*r, rat; h, human; n.d., not determinated. ^∗^*In vivo* studies.*

In the adrenal cortex of rat and human, endocrine cells in all three layers express NPB and NPW as well as NPBWR1 and NPBWR2 (Hochol et al., 2004, 2006; Mazzocchi et al., 2005). *In vitro* experiments show that both peptides are able to stimulate basal glucocorticoid, corticosterone, or aldosterone secretion from dispersed or cultured adrenocortical cells depending on species (Mazzocchi et al., 2005; Hochol et al., 2006). Parenteral application of NPB and NPW leads to rise of plasma concentration of corticosterone (Hochol et al., 2006). In the adrenal medulla, NPW derived from chromaffin cells could interact with adrenocortical cells in a paracrine or endocrine manner (Seki et al., 2008). Direct effects of NPB or NPW on secretion of catecholamines from chromaffin cells are not known yet.

In the rat endocrine cells of Langerhans islets, the NPB/NPW signaling system may have paracrine modulatory functions of islet cells. Administration of NPW but not NPB transiently lower blood insulin levels (Rucinski et al., 2007).

In the rat thyroid and parathyroid glands, these peptides may control secretory activity, while administration of both NPB and NPW evoked a marked rise in the blood level of parathyroid hormone but only NPB not NPW application was able to increase plasma level of thyroxin (Hochol et al., 2006).

In the rat ovary and testis, NPB/NPW signaling system has been shown to stimulate release of estradiol and testosterone, suggesting its role in the regulation of gonadal hormone secretion (Hochol et al., 2006).

In the rat pituitary gland, prolactin secretion is elevated and growth hormone release inhibited by central administration of NPW (Baker et al., 2003). Vasopressin and oxytocin neurons projecting to the posterior pituitary showed responsivity to NPW, although some were hyperpolarized and others depolarized (Price et al., 2009).

In isolated rat adipocytes, both NPB and NPW reduce leptin expression and secretion and increase lipolysis. However, only NPB not NPW enhanced secretion of resistin (Skrzypski et al., 2012). These effects were mediated by NPBWR1 and suggest involvement in glucose and lipid homeostasis (Skrzypski et al., 2012). The exact cellular mechanism remains to be determinated.

In isolated arterial smooth muscle cells as well as in transfected HEK 293 cells, NPW modulates the Ca_v_1.2 current via NPBWR1 though the PLC/PKC pathway, which leads to changes in arterial tone. This mechanism may be involved in the development of vascular hypertension (Ji et al., 2015).

From the aforementioned studies, it is clear that the NPB/NPW signaling system has a significant effect on the regulation of endocrine functions. The effect of these peptides on endocrine cells *in vitro* or on the function of endocrine glands *in vivo* was studied. In particular, *in vivo* experiments demonstrate the ability of each peptide to function as hormones. At the same time, the paracrine effect of NPW was also demonstrated in the vessel wall.

## NPB/W Signaling System in the Cardiovascular System

A number of studies have demonstrated the presence of mRNAs of individual members of the NPB/W signaling system in several organs. In most cases, these data were obtained by RT-PCR, so nothing indicates the tissue distribution of this signaling system. Such a large distribution may indicate that the source and/or target could be a certain types of cells/tissues present in all organs. These include, for example, blood vessels or the cells of the immune system. It has now been discovered that smooth muscle cells have on their surface NPBWR1, through which NPW increases the flow of calcium into the cell and thus regulates vascular tone. The significance of this signaling system in vascular tone regulation is also indicated by the fact that hypertensive rats have a decline in NPW expression in the blood vessels(Ji et al., 2015), which probably serves as a compensatory mechanism to reduce Ca_v_1.2 channel function in the pathophysiology of hypertension (Ohya et al., 1993). On the base of these results, participation of NPW in pathophysiology of hypertension was suggested (Ji et al., 2015). Additionally, there is an evidence suggesting involvement of NPW in blood pressure regulation by a central modulatory mechanism. Yu et al. (2007) demonstrated that central application of NPW lead to significant rise of blood pressure and HR. This effect may be mediated by activation of the orexin-producing neurons located in the lateral hypothalamic area, the same site where the presence of NPW-IR nerve fibers was described (Takenoya et al., 2010a). It has been found that blocking the function of orexin-producing neurons results in omission of the effect of centrally administered NPW on blood pressure (Pate et al., 2013b).

While assessing NPB and NPW functional similarities, we could observe a similar functional behavior with another neuropeptide – neuropeptide Y (NPY). NPY is expressed in similar parts of the CNS as NPB and NPW (Chronwall et al., 1985). Additionally, its major function is the regulation of energy metabolism including the regulation of food intake (Balasubramaniam, 2002; Magni, 2003). At the same time, however, this peptide is also widely distributed within the body including neurons and non-neuronal tissue and significantly contributes to the regulation of cardiovascular system functions (Balasubramaniam, 2002). Another parallel trait or characteristic between NPW and NPY is their highly conserved sequence during evolution (Larhammar, 1996). Whether NPW exerts similar functions in cardiovascular regulation as NPY does remained to be elucidated. Information concerning to exact tissue distribution of NPB/NPW signaling system within the heart and other peripheral tissues as well as some functional studies are needed in order to confirm the assumption.

## NPW Expression During Development

Currently, there is limited information concerning to function of NPB and NPW during development. Motoike et al. (2015) studied expression of NPW in the postnatal mouse dorsomedial hypothalamus. They demonstrated robust, but transient, expression of NPW mRNA, which was highest at about P14 and suggest that such increase of the expression of this time may be related to the important transition from milk suckling to solid food intake. NPW expression also exhibits developmental changes in the stomach, where it is progressively increased with age until reaching adulthood (Mondal et al., 2008). Both these finding suggest important role of NPW in the development of feeding regulation.

## Conclusion

The NPB/W signaling system was described over/during past two decades, and since then many studies have been devoted to it. Their location was also studied thoroughly and confirmed in the CNS; however, the information related to distribution in peripheral organs and tissues is still sporadic. Although the expression of mRNAs of individual members of this signaling system has been demonstrated in most of the organs, their exact localization at the cellular level is still unknown in many cases. Moreover, its localization and function in endocrine glands have been studied in more detail, with results showing the involvement of this system in a number of regulatory mechanisms. Nevertheless, so far, there has not been any satisfactorily explanation regarding the presence of this signaling system in other organs and tissues.

Concerning to cardiovascular system, strong evidence exists suggesting involvement of NPB/NPW signaling system in regulation of blood pressure on the central as well as peripheral level. Information about tissue localization and function of this system within the heart needs to be elucidated. Expression of the signaling system members’ mRNA has already been demonstrated in the heart of several species (Fujii et al., 2002; Brezillon et al., 2003; Fang et al., 2015; Bu et al., 2016; Yang et al., 2018).

## Author Contributions

The author confirms being the sole contributor of this work and approved it for publication.

## Conflict of Interest Statement

The author declares that the research was conducted in the absence of any commercial or financial relationships that could be construed as a potential conflict of interest.
